# A Post-Processing Method Based on Radial Basis Functions for the Fast Retrieval of the Strain Field in Digital Image Correlation Methods

**DOI:** 10.3390/ma15227936

**Published:** 2022-11-10

**Authors:** Corrado Groth, Andrea Chiappa, Stefano Porziani, Marco Evangelos Biancolini, Emanuele Marotta, Pietro Salvini

**Affiliations:** Department of Enterprise Engineering, University of Rome “Tor Vergata”, 00133 Rome, Italy

**Keywords:** RBF, FEM, large displacements, digital image correlation, strain measurement, meshless

## Abstract

Digital image correlation methods allow the determination of the displacement (and thus the strain) field of a target by picture comparisons, without the application of strain gauges or other invasive devices. Homologous sites are mapped from the undeformed to the deformed configuration, and displacements retrieved at a cloud of points in a scattered fashion. Radial basis functions (RBF) offer a rapid and reliable tool to post-process on-the-fly data from image correlation, in order to compute deformations directly without the need for generating a numerical grid over the measurement points. Displacements and associated strains can be computed only where desired, tracking automatically only the most reliable features for each image. In this work, a post-processing strain evaluation method for large displacement problems, based on RBF and the Green–Lagrange tensor, is presented and demonstrated for several test cases. At first, the proposed method is adopted on a set of artificially generated pictures, demonstrating a faster convergence with respect to FEM even when few points are used. Finally, the approach is applied to cases for which experimental results are available in the literature, exhibiting a good agreement.

## 1. Introduction

The digital image correlation technique is gaining increasing interest due to the possibility of measuring the strain field of a target without the application of any invasive device in situ, only resorting to the comparison of digital images taken during load application [1]. Although other strategies are possible [2], the painted speckle technique is the easiest way to perform the correlation strategy since it requires a simple surface preparation and cheap equipment [3]. The painted speckles are a scattering of irregular spots that, in digital images, correspond to clusters of colored or grey pixels. The tracking of such reference points allows reconstruction of the evolution of a target across images. Starting from the original work by Peters and Ranson [4], many authors propose their approaches to this goal: bicubic spline interpolation [5], wavelet transformations [6], and techniques based on FEM representation of displacements [7] just to cite a few. In [8], the modal pursuit method is proposed, which is able to gain large displacements from the comparison of only two pictures: before and after load application.

Digital image correlation methods are able to track corresponding points over a series of images taken at progressive steps of load application. Output data from correlation methods are the displacement given at scattered points on the target domain, with the consequent need for an underlying base to compute the strain (and stress) field. A computation base can be the numerical grid obtained, taking the observation sites as FEM nodes, with elements connecting them. Such a strategy suffers from shortcomings due to the difficulty of obtaining good-quality, regular-shaped elements from any arrangement of points located over the target image. Moreover, it is hard to achieve a rich tracking with a high number of tidily arranged points, resulting in a coarse grid showcasing low fidelity and poor resolution on the peak strains.

Meshless methods [9] from this point of view offer an alternative way to elaborate granular data from comparison of images, avoiding the task of mesh generation. The basic idea behind this class of techniques is that approximation relies directly on nodes, which often contribute to the final result on a distance basis. Major advantages of mesh-free methods are the smoothness and continuity of the output, often analytically guaranteed, as well as their natural scalability up to the smallest distances, other than the above-mentioned independence from a numerical grid. Lucy [10] was the first to use a meshless strategy to study a complex astrophysical phenomenon, simply considering mutual interactions in a set of points. In subsequent years, the idea initially proposed by Lucy aroused the interest of other authors, which further developed the original method and extended its range of application [11,12,13,14]. The diffused approximation [15] and the partition of unity [16] methods share the basic idea of the decomposition of a unique domain in several subdomains, with the continuity of the output field recomposed through a partial superposition of the subdomains. Other methods combine a meshless approximation base with a differential equation solution technique to obtain a complete analysis frame, able to solve a system and interpolate the solution everywhere in the domain [17,18].

In this paper we propose the synergic use of a tracker based on the Kanade–Lucas–Tomasi algorithm and Radial Basis Functions (RBF), a special class of mathematical functions able to meshlessly interpolate data available at points, for the strain field retrieval on digital images. The proposed method can be intended as a post-processing step in which the advantages connected with the use of RBF, such as their ability to derive a smooth analytical form for displacements known only at points, can be exploited in an automated fashion given a set of images before and after load application as input. RBF prove to be reliable and agile in reconstructing the strain field starting from spotted displacement data, providing a continuous approximation on a node basis without the need for domain tessellation. To demonstrate the approach, at first the method is tuned and compared to known results on a numerical testcase, feeding the proposed tool with images of the undeformed and deformed FEM mesh. In this way it is possible to quantify the absolute error introduced by the tracking and reconstruction algorithms, tuning the number of RBF points required by the method in order to obtain a faithful strain field representation. Then two empirical cases, previously tackled in [8] by means of the modal pursuit method, are considered and compared to the literature data.

## 2. Radial Basis Functions

Radial basis functions (RBF) [19] were initially introduced in the context of multi-dimensional interpolation. They addressed the need for a reliable interpolation technique, able to process spotted data given at an irregular distribution of points. An RBF interpolant accounts for all the data supplied, with the guarantee of the exact retrieval of a given value at the point where it is assigned in input, without suffering from the instability typical of multi-terms polynomial supports.

In general terms, input data are in the form of values given at certain points **x_i_** (source points) in the m-dimensional domain. The RBF interpolator approximates the spotted distribution of information with a continuous analytical function, whose value *s*(**x**) at a target point **x** is given as:

(1) $$s\left(x\right)=\overset{N}{\underset{i=1}{\sum }}\gamma_{i}\varphi \left(\|x-x_{i}\|\right)$$

where: *N* is the number of source points, *γ_i_* are the weights deriving from the above-mentioned retrieval condition and *φ* are the radial bases. The behaviour of the interpolant between the points depends upon the chosen kernel, i.e., the particular form of the selected radial function. Table 1 reports typical RBF kernels, where $\;r=\|x-x_{i}\|$ and ϵ is a shape parameter [20], which should cope with the average grid spacing. As regards the Generalized MultiQuadratic (GMQ) kernel, *R* and *q* are parameters subject to the user’s choice. It is evident that the GMQ kernel can assume all the forms reported in Table 1, with the exception of the thin plate spline and the Gaussian. It is worth noticing that a radial basis is a function of the Euclidean distance $\left\|x-x_{i}\right\|$ of two points in a multidimensional space that gives in output a simple scalar, reducing the dimension of the problem.

Indicating with g_i_ the input values associated to the source points and with **g** the vector collecting them in a row, the vector **γ** of weights has to satisfy the condition:

(2) Mγ=g

which gathers in a matrix and forms the equations as Equation (1) written for all the given points of the system, with **M** containing the radial bases computed at each source point. Upon the inversion of the square matrix **M**, the weights *γ_i_* are known.

Often a polynomial supplement *h*(**x**) is added at the end of the summation in Equation (1) to enhance the potency of the interpolant, which acquires the capability to reproduce exactly those functions of the same form of *h*(**x**). In this case also the polynomial coefficients are unknown, and the system assumes a different form from that in Equation (2). A condition of orthogonality [21] between the weights *γ_i_* of the radial bases and the monomial terms constituting the polynomial supplies additional constraints to solve the enlarged system. In the case of a linear polynomial in a 2D space:

(3) $$h\left(x\right)=\beta_{1}+\beta_{2}x+\beta_{3}y$$

The system (2) assumes the form:

(4) $$\left[\begin{array}{cc} M & P\\ P^{T} & 0 \end{array}\right]\left(\begin{array}{c} \gamma \\ \beta \end{array}\right)=\left(\begin{array}{c} g\\ 0 \end{array}\right)$$

where **P** is the constraint matrix deriving from the orthogonality condition:

(5) $$P=\left[\begin{array}{ccc} 1 & x_{1} & y_{1}\\ 1 & x_{2} & y_{2}\\ \vdots & \vdots & \vdots \\ 1 & x_{N} & y_{N} \end{array}\right]$$

The degree of the polynomial term depends upon the adopted radial basis, and a table summarizing the possible associations can be found in [20].

RBF interpolation also allows the handling of vector fields, in this case each component of a vector is treated individually. Taking the example of a 2D vector field:

(6) $$\{\begin{array}{c} s_{x}\left(x\right)=\overset{N}{\underset{i=1}{\sum }}\gamma_{i}^{x}\varphi \left(\left\|x-x_{i}\right\|\right)+h_{x}\left(x\right)\\ s_{y}\left(x\right)=\overset{N}{\underset{i=1}{\sum }}\gamma_{i}^{y}\varphi \left(\left\|x-x_{i}\right\|\right)+h_{y}\left(x\right) \end{array}$$

Further details on RBF can be found in [22].

RBF prove to be extremely versatile tools, since their possible application fields are legion: from neural networks [23] to computer graphics (surface reconstruction [24]), and from mesh morphing [25,26] to image analysis of deformations [27] and data transfer [28]. RBF mesh morphing has been employed for several applications, from FSI coupling [29] to genetics [30], evolutionary optimizations [31], and advanced modelling [32]. RBF have been employed successfully as a post-processing tool: in [33] the authors proposed a method to improve stress results from coarse FEM models via RBF interpolation of nodal displacements. In [34] and in [35], FEM accuracy was enhanced with RBF interpolation of results enriched with balance equations. The Kansa method [36,37] allows the integration of differential equations expressed with RBF fields: the solution of problems starting from boundary conditions [38,39], as well as the completion of partial results obtained by FEM [40], are both possible applications of this strategy. This approach was also shown in [41,42] to compute strains from displacement field.

## 3. The Method

**Figure 1:**
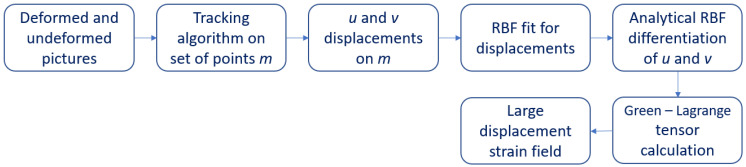
Flow chart of the proposed method.

The proposed method, as shown in the flow chart in Figure 1, takes in the input *x* and *y* components of displacements (indicated with *u* and *v* in the remainder) at scattered points over a 2D domain, which coincides with the object of deformation. Displacements derive from the difference between the current position of a point, when load is completely applied, and its former position, when no load is acting. To reduce the error introduced by the tracking algorithm, a set of pictures taken while increasing the load can be employed, but it is not mandatory. This characteristic can be exploited to obtain a real-time strain retrieval by providing the picture stream, for example taken from a camera, to the proposed method. Two independent RBF interpolants for the components *u* and *v* of the displacement are introduced. Under the assumption of small rotations and deformations, the components of strain simply yield as a combination of the first order derivatives of the displacements:

(7) $$\varepsilon_{x}=\frac{\partial u}{\partial x}\;\varepsilon_{y}=\frac{\partial v}{\partial y}\;\gamma_{xy}=\frac{\partial u}{\partial y}+\frac{\partial v}{\partial x}$$

Considering a GMQ kernel with a polynomial supplement, first order derivatives can be easily calculated:

(8) $$\frac{\partial \varphi \left(r\right)}{\partial x}=q{\left(\epsilon^{2}r^{2}+R^{2}\right)}^{q-1}2\epsilon^{2}\left(x-x_{i}\right)+\beta_{2}\;\frac{\partial \varphi \left(r\right)}{\partial y}=q{\left(\epsilon^{2}r^{2}+R^{2}\right)}^{q-1}2\epsilon^{2}\left(y-y_{i}\right)+\beta_{3}$$

and inserted in Equation (7) to compute strain values. The described procedure takes in input point-wise data and does not require any grid to produce output results. RBF allow the translation of this transformation known only at points in a derived field through analytical, and thus exact, differentiation. This simple feature represents an important advantage with respect to other methods, since points can be meshlessly tracked only where required in real time. Moreover, by not having stiff constraints in terms of nodal coordinates to be tracked, the quality of the reconstructed displacement field can be kept high, following only the points for which the most reliable tracking can be assured on a case-by-case scenario.

In the following applications we adopted R=0.1, q=1.5 and ϵ=1, with no polynomial supplement. To tackle problems dealing with highly deformable specimens, moreover, we proposed in this paper an approach to study large displacements taking advantage of the continuous analytical field obtained by means of RBF, circumventing all the shortcomings of a FEM-based approach such as the preparation and handling of a non-linear analysis.

Taking into account large displacements, the Green–Lagrange tensor [43] offered a co-rotational basis for strain evaluation. The deformation gradient **F** for the points on the plane assumed the form:

(9) $$F=\left[\begin{array}{cc} \left(1+\frac{\partial u}{\partial x}\right) & \frac{\partial u}{\partial y}\\ \frac{\partial v}{\partial x} & \left(1+\frac{\partial v}{\partial y}\right) \end{array}\right]$$

in which derived quantities were obtained by means of (8).

The Green–Lagrange tensor of the transformation in object can be written from (9) as:

(10) $$E=\frac{1}{2}\left(F^{T}F-I\right)$$

from which the desired quantities of the deformation can be extracted everywhere in the space, being this tensor continuous and available analytically. Cartesian strains can be calculated as:

(11) $$\varepsilon_{x}=\sqrt{2E_{11}+1}-1\;\mathrm{and}\;\varepsilon_{y}=\sqrt{2E_{22}+1}-1$$

**Figure 2:**
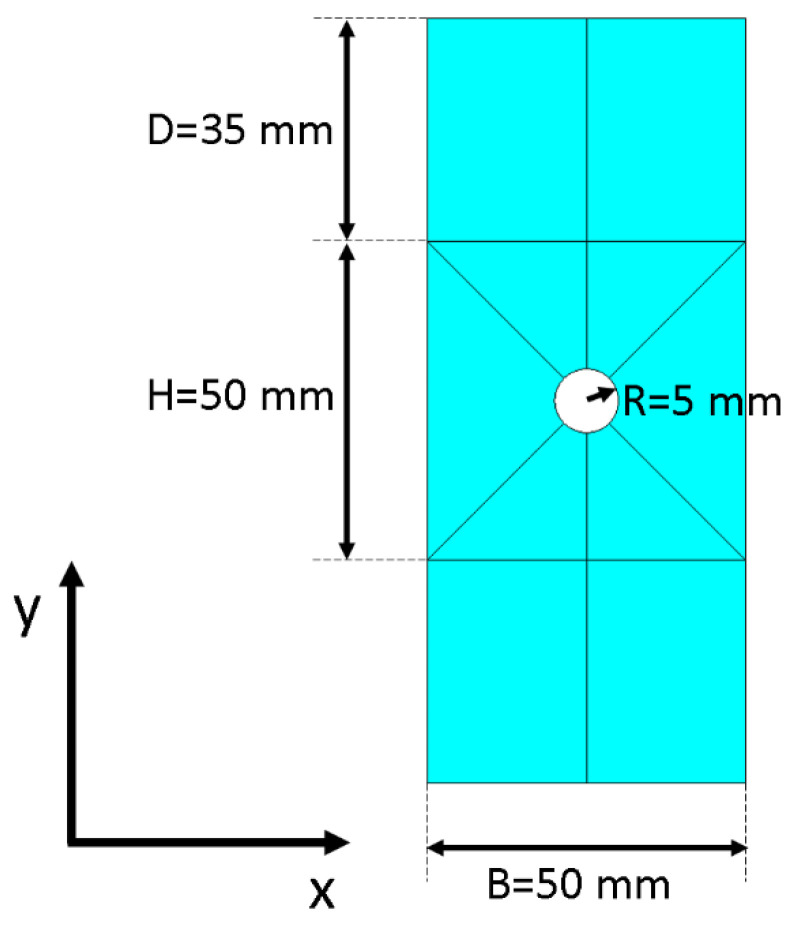
Geometry of the drilled plate.

Equalities in (11) derived from the comparison of the Green–Lagrange tensor as obtained in (10) with the well-known form of summation of tensor products often recurring in the literature ([43]). To demonstrate the method, it was first tested against a set of artificially generated pictures. A Kanade–Lucas–Tomasi (KLT) tracking algorithm [44,45] implemented in Matlab was employed to retrieve the scattered point-wise displacements, and the reconstructed continuous strain field achieved by the method compared to numerical results. A plane stress FEM model of a plate with a central hole under an imposed tensile load of 1080N was employed for the purpose. Figure 2 sketches the geometry of the plate: a central square (B × H) with a hole of radius R in the middle and two buffer rectangles (of height D) above and below to attain a zone of constant stress at a certain distance from the singularity when a tensile load was applied along *y*. The out-of-plane thickness was 1.5 mm.

Since the focus of this case was to test the method against a large displacement scenario, demonstrating its ability in faithfully retrieving the strains—once fed with the correct displacements—and to distinguish them from the pure rotational contribution, the type of material model employed was of secondary importance and a perfectly elastic material was used. The goal of this application was not to reproduce exactly the experimental behavior of the specimen, but to generate a test case complex enough to challenge the proposed method, regardless of the source of the displacements used. For this reason, the Young modulus used was 70 MPa with a Poisson’s ratio equal to 0.46.

**Figure 3:**
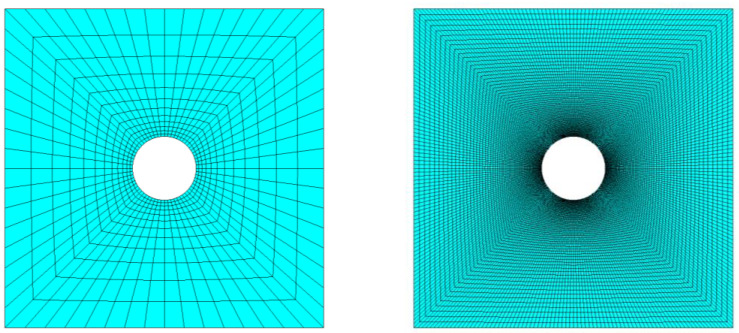
The central square of the plate featuring the coarsest (**left**) and finest (**right**) levels of discretization on the biased mesh.

As a first step, convergence obtained with RBF interpolation was compared against FEM. A series of analyses was run having as an object the model of the plate with an increasing level of refinement. Figure 3 compares for the central square of the plate the coarsest and the finest levels of discretization employed for the convergence test, having generated a meshing increasingly refined near the stress concentration with parabolic 8-nodes SHELL281 elements, well-suited for large strain applications.

**Figure 4:**
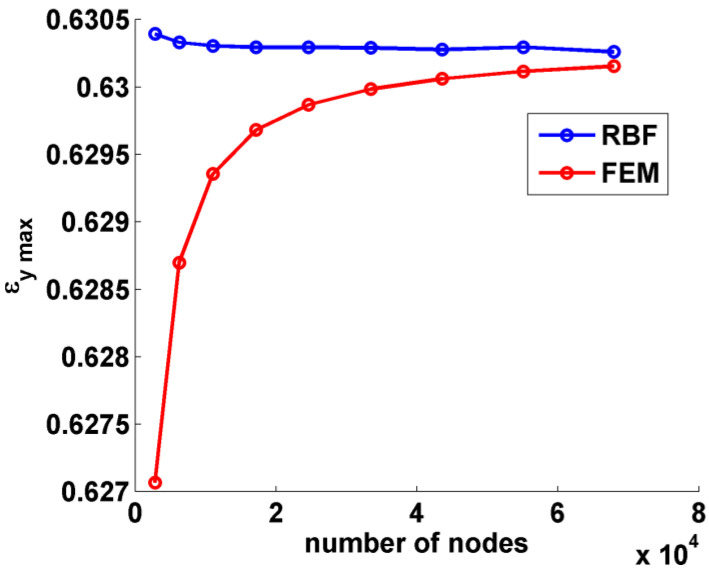
Convergence trends for the RBF post-processing method (blue) and FEM (red) on the mesh refined near the hole.

At the end of each FEM analysis, nodal displacements in the proximity of the hole supplied input data for the RBF post-processing method. Figure 4 reports the maximum value of *ε_y_* obtained both with FEM and after differentiation of the RBF field for each value of the discretization level, represented with the number of nodes.

As already pointed out by the authors in [33], the convergence behavior of RBF was much more stable than that exhibited by FEM: the maximum value of ε_y_ retrieved with RBF remained practically unchanged over the number of nodes, with FEM results approaching from below. With a relatively small number of nodes, however, in the order of 10^4^, the results obtained with both methods could be considered very good. In must be noted that the value of deformation studied was very high, in the order of 60%.

Having demonstrated that, with a proper mesh, refined near the singularity, FEM and RBF methods led to similar results, a second test was carried out on an unbiased mesh, in which nodes were equally spaced radially with respect to the hole.

**Figure 5:**
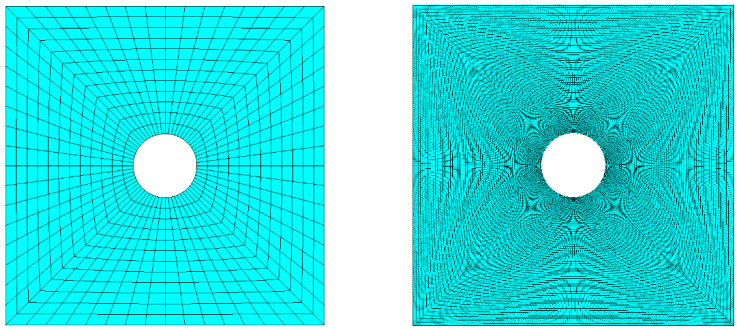
The central square of the plate featuring the coarsest (**left**) and finest (**right**) levels of discretization on the unbiased mesh.

This experiment was significant since, in several cases, it was impossible to retrieve displacements with a proper accuracy near the higher gradients of deformation using digital image correlation methods, making it necessary to rely on points tracked with an equidistant pattern. In addition, in this case a series of analyses was run, increasing mesh refining as shown in Figure 5.

**Figure 6:**
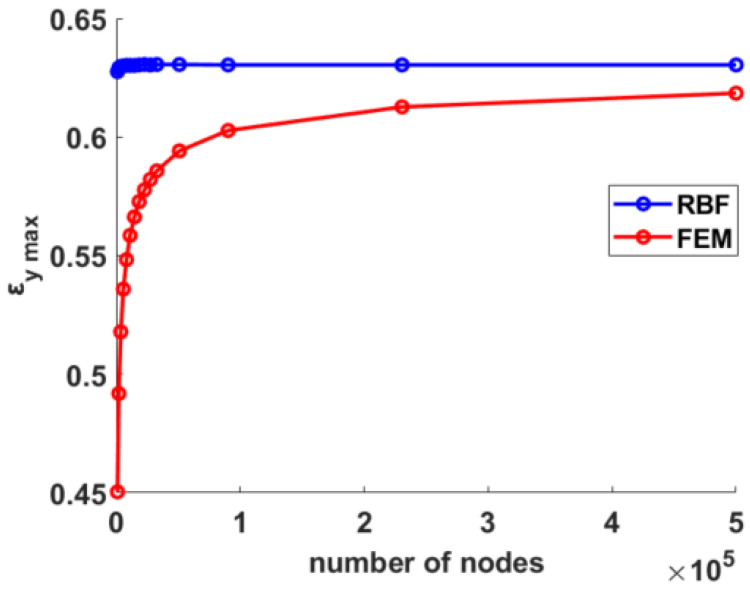
Convergence trends for the RBF post-processing method (blue) and FEM (red).

Nodal displacements retrieved on the FEM mesh were fed to the RBF method for each case and results in terms of maximum strain along the y direction plotted in Figure 6. Once again, the RBF results demonstrated surprising stability, remaining substantially unchanged over the range of tested mesh refinements. FEM values, on the other hand, even if approaching to the correct value from below, similarly to the previous case, suffered convergence problems and encountered difficulty reaching it. Tests were carried out on up to half a million nodes, and yet the two trends remained separate in the considered range of mesh refinement; nonetheless they give the impression of meeting for a degree of discretization far beyond the limit here considered, and specifically to the value of 0.63 retrieved on the previous mesh refined near the hole.

This convergence behavior is one of the strengths of the proposed method, since a good-quality strain reconstruction can be achieved using RBF even when few points are tracked, in contrast to methods based on FEM shape functions that do require a finer discretization of the domain. Moreover, to obtain satisfactory results using FEM methods, an ad hoc mesh must be generated with proper refinements near singularities and stress raisers for each case, requiring a complex and time-consuming procedure of pre-processing. Conversely, the proposed RBF method, while providing very good results even when a small number of points were available as shown in the first example, did not require a case-specific point seeding, being able to obtain excellent results even with an equidistant mesh, as shown in the second example. This characteristic is a key feature of the method, which not requiring a pre-processing stage and being problem agnostic can be directly applied to a new case, retrieving results in real time.

To demonstrate this, the same example of the drilled plate was employed to test the whole proposed workflow, feeding the pictures of the numerical grid into the proposed tool as if they were the result of an experimental campaign, having at the same time the ability to compare results to those directly obtained using a fine numerical grid in the FEM environment. The tessellation of the surface into elements allowed the creation of a red and blue irregular chessboard, with the same level of discretization as that of the numerical grid. Two mesh sizes were explored, and for each one the number of the tracked points was parameterized in order to understand their influence in the strain retrieval process, comparing the results with those achieved with a fine FEM grid.

**Figure 7:**
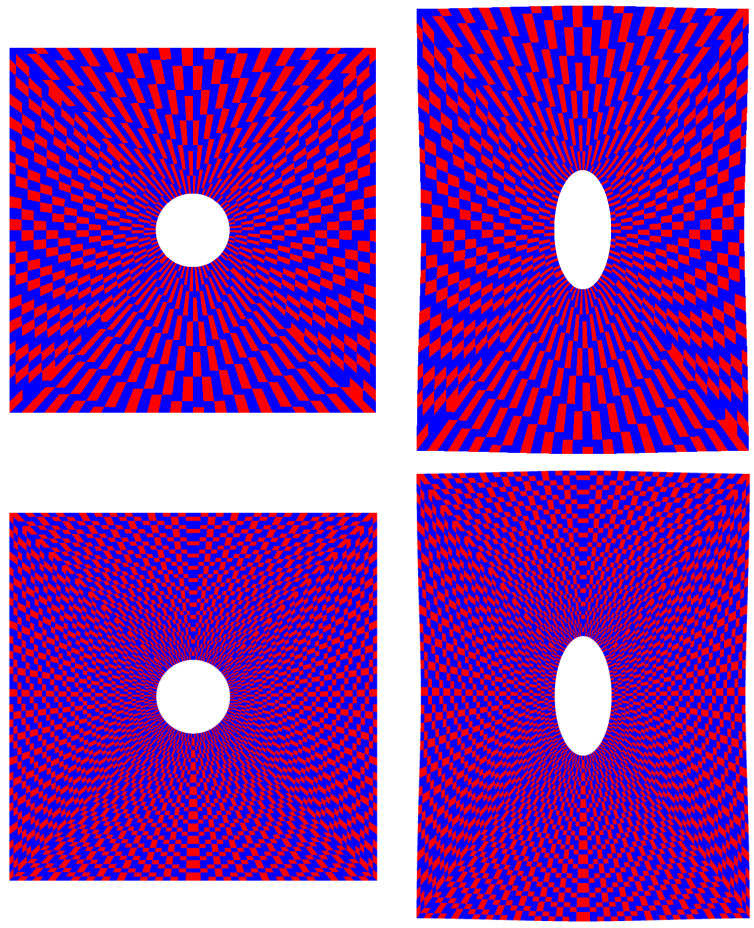
Coarse (**top**) and fine (**bottom**) meshes in undeformed (**left**) and deformed (**right**) configuration.

In Figure 7, the coarse and fine meshes employed for this study, counting respectively 1824 and 7104 nodes, are shown in their undeformed and deformed configuration resulting from load application.

**Figure 8:**
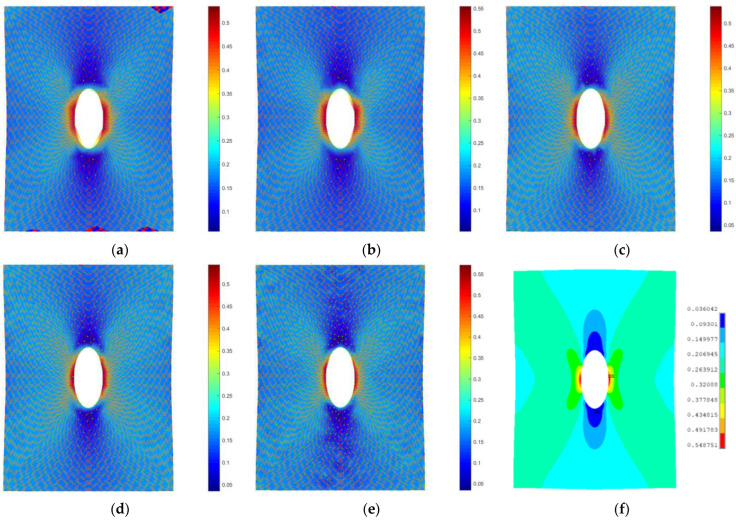
Strain field computed by means of proposed method on the fine grid increasing the number of tracked points: (**a**) 299, (**b**) 407, (**c**) 582, (**d**) 794, (**e**) 1299 and comparison to FEM results with 7104 nodes (**f**).

In Figure 8, the strain fields computed by means of the proposed method on the fine grid (using the pictures from the second row of Figure 7) are shown, superimposed on to the image of the deformed specimen, progressively increasing the number of points tracked. Reference FEM contours computed on the full fine grid are shown in Figure 8f. It must be noted that, being a meshless method, the proposed approach supplies everywhere in the space a continuous value of deformation that can be retrieved where desired. To plot results conveniently, contours are applied on a support grid automatically generated by means of a Delaunay triangulation on the tracked points. For this reason, in the first two pictures of Figure 8, in which few points are used, the central hole of the specimen is approximated by a polygonal chain that is only aesthetic and does not affect the solution.

**Figure 9:**
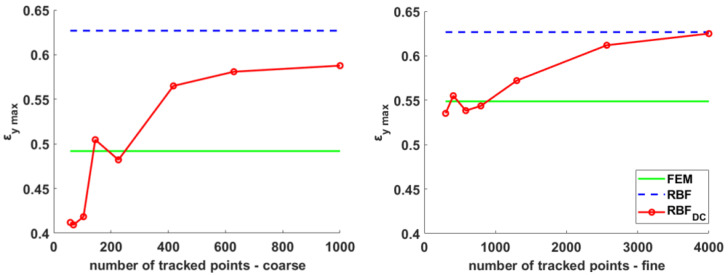
Maximum strain for the coarse (**left**) and fine (**right**) meshes computed using the proposed method while increasing the number of tracked points (red) and reference values on the same grid obtained with FEM (green) and RBF (dashed) computation.

Results in terms of maximum deformation along the y direction are shown in Figure 9, in which the values achieved using the proposed method (red lines) are plotted against the number of tracked points for both the coarse (left plot) and fine (right plot) meshes, counting 1824 and 7104 nodes. Such values are compared to the reference results obtained on the same mesh using FEM (green) and those achieved by supplying its displacements to the RBF post-processing method (blue dashed line). As already shown in Figure 6, for a broad range of mesh densities, the RBF results remained tendentially unchanged, while FEM strains approached to a convergence value from below increasing mesh refinement. The quality of the results achieved with the proposed method (RBF_DC_), as expected, depended on the number of points employed for tracking, demonstrating however a better convergence to finer results with regard to FEM even when employing the method on coarser meshes. It must be noted that the proposed approach was able to retrieve the large displacement strains associated with a multi-axial in-plane deformation. For reasons of space, however, we addressed only cases in which a prevalent deformation along the y axis was applied, similarly to what happens when dealing with a traction machine. For this reason, being the strain along the *y* direction prominent, the *ε_x_* was not plotted. In the next section, however, the comparison between two literature cases and the proposed method was carried out using, as a term of comparison, the equivalent strain, that took into account the strains along both the *x* and *y* axes.

## 4. Experimental Testing

After the preliminary setup of the method with controlled images, the same experimental cases previously presented in [8] were considered. They were two instances of load application to drilled specimens, where strong strain concentrations were present.

The first one was a polyethylene structure with a hole in the middle resembling the numerical tests shown before. The sample was highly stretched along the vertical direction. The total number of points employed for tracking was 1528.

**Figure 10:**
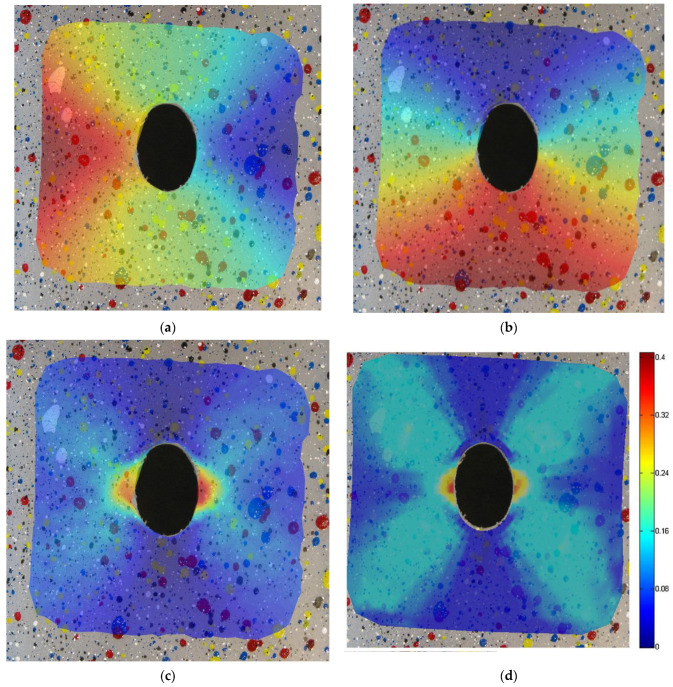
Horizontal (**a**) and vertical displacements (**b**) for the drilled experimental sample under traction. In (**c**) the equivalent strain values retrieved by means of the proposed method are compared with those in the literature (**d**).

**Figure 11:**
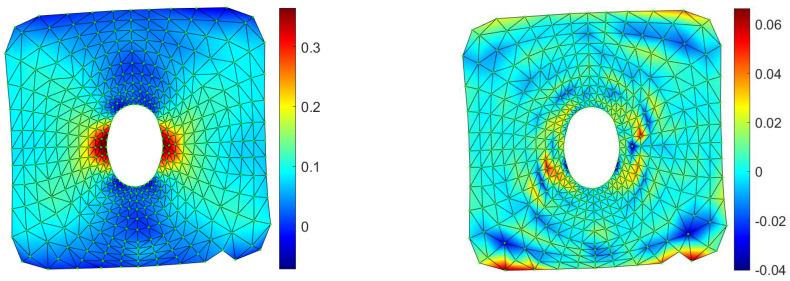
FEM contour map of the strain along the y direction for the drilled case (**left**) and its difference with respect to the proposed method (**right**).

**Figure 12:**
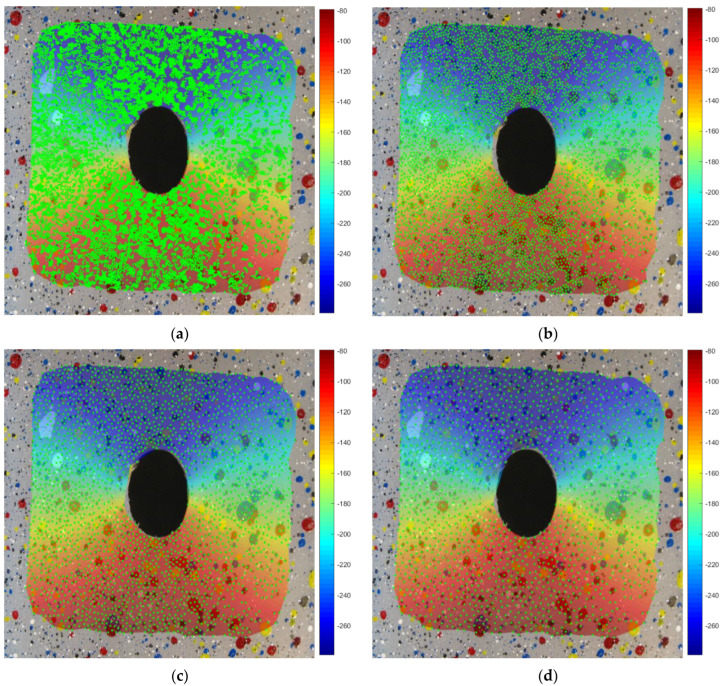
Vertical displacements computed by RBF varying the number of tracked points. (**a**) 10217 points, (**b**) 4149 points, (**c**) 2343 points, (**d**) 1528 points.

In Figure 10, the contour maps computed with the proposed method of the horizontal and vertical displacement in pixel are shown, together with the comparison of the equivalent strain with the one available in the literature. Both methods captured a strain peak slightly higher than 40% at the hole sides, with a percentage error of the proposed method of 1.47% with respect to the literature experimental data [8]. In Figure 11, the strain field, computed on a FEM model reproducing the experimental case, was compared to the proposed method, showcasing a maximum error, in terms of *ε_y_*, equal to 0.063. It is important to stress that in some specimens in which large deformation and stretching occurs, it is difficult to achieve high-quality tracking, especially near large deformation areas. In these cases, RBF are able to fill the gap, interpolating smoothly the missing information from point to point thanks to the analytic representation they provide. In Figure 12, the vertical displacements for the drilled polyethylene specimen are reconstructed, changing the number of tracked points. It can be appreciated how the contour maps and values remain substantially unchanged, even if the density of points near the most distorted area on the side of the hole is far smaller with respect to other parts of the specimen.

**Figure 13:**
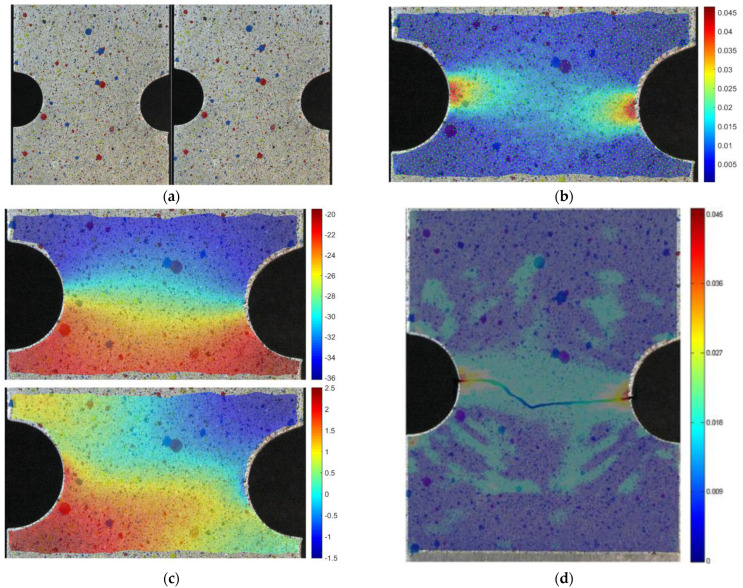
Unloaded and loaded aluminum specimen (**a**). Vertical and horizontal displacements due to loading (**c**) and equivalent strain maps for the aluminum alloy sample using RBF and tracking (**b**) and using modal tracking (**d**).

The second instance is an aluminum alloy specimen, with two half-holes at the sides, not perfectly aligned along the horizontal axis. In Figure 13a, the original and deformed geometries are shown in the left and right pictures respectively. In (c), the vertical (top) and horizontal (bottom) displacements are shown as interpolated by means of RBF from tracked points. it is interesting to notice how the proposed method catches how the horizontal displacements are not symmetrical due to the misalignment of the notches along the horizontal axis. The equivalent strain distribution obtained by means of point tracking and RBF is shown in (b), overimposed on the deformed specimen, calculated with 2029 points. Results can be compared to those available in the literature, as shown in (d), in terms of strain values and distribution. For both methods, the maximum value of equivalent strain is slightly higher than 4.5%, with an error of the RBF method of 1.75% with respect to the data available in the literature [8].

**Figure 14:**
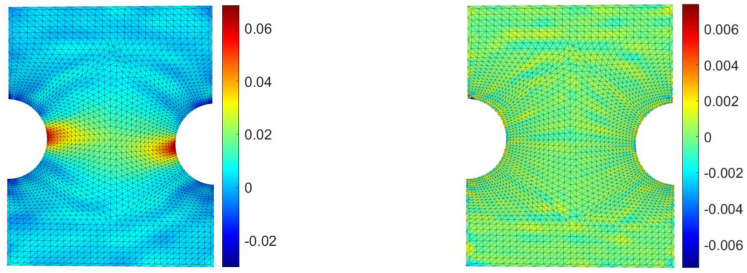
FEM contour map of the strain along the y direction for the aluminum specimen (**left**) and its difference with respect to the proposed method (**right**).

In Figure 14, the comparison between the RBF approach and a FEM model reproducing the experimental case is shown on the right picture, in which the difference between both methods is plotted on the numerical grid for the strain along the y axis. The maximum strain difference, located far from the hot-spot, is equal to 0.0066.

The proposed post-processing method proved to reconstruct accurately the strain field starting from two images pertaining to the unloaded and loaded states of a specimen. The accuracy of the RBF interpolant in capturing the position and the value of a strain peak is comparable to that exhibited by the well-established FEM, based on shape functions. The great advantage of the described strategy is that it eliminates the tedious and nontrivial task of building a quality grid from nodal positions, being RBF interpolation on a node-basis, substituting this task with point seeding on images and their direct tracking by means of a KLT algorithm. This process can be automated and performed in real time, distributing points only where required and focusing on the areas of interest.

With respect to FEM-based methods, a digital image correlation routine based on RBF guarantees higher quality and faster convergence to results, and for a very low number of points. As a downside of the method, and possible starting point for future research, we must note that the displacements of the tracked points are taken as they are by the RBF algorithm and, since the quality of the strain reconstruction closely depends on their reliability, a possible tracking error has a huge influence and cannot be identified at the current time. While a first check on the tracking quality is already carried by performing it back and forth, keeping only the points within a given tolerance, we foresee in the future computing the reliability of each point using a physical approach based on structural mechanics. A further disadvantage of the presented method is the difficulty in considering the third axis, meaning that it is suitable only to be applied for flat images in which the deformation does not involves an important displacement along the normal to the plane.

## 5. Conclusions

In this work, a post-processing method for the strain retrieval in large displacement problems using digital-correlation techniques was proposed. The method is based on the use of Radial Basis Functions (RBF) to represent the displacement field, available in a point-wise fashion and obtained by means of a tracking algorithm based on the known Kanade–Lucas–Tomasi approach. RBF were able to convert the point-wise data in a continuous field that could be analytically differentiated to obtain strains where desired, using the Green–Lagrange tensor for large displacement problems. The proposed method demonstrated that it provided high accuracy in retrieving the position and value of the peaks, with a quality comparable to that of a highly refined FEM based on shape functions. Contrarily to a FEM approach, the proposed method allowed automation of strain retrieval, making possible a real-time calculation, being meshless and not requiring a case dependent meshing but an equidistant RBF point seeding. Moreover, this guaranteed faster convergence with respect to FEM, requiring a lower number of tracked points to obtain comparable results.

In summary, the proposed method:

- retrieves the values of strain from large displacements in a fast and accurate manner;
- is easy to apply because it does not require a connectivity organizing the nodes into elements;
- features a faster convergence with respect to FEM.

However

- is sensitive to local oscillations introduced by noise because differentiation is point-wise and not averaged on an element basis;
- it is suitable to be used in planar cases.

## Figures and Tables

**Table 1:** Most common radial basis functions.

RBF	*φ*(*r*)
Spline type (Rn)	$r^{n},\;n\;odd$
Thin plate spline (TPSn)	$r^{n}\log\left(r\right),\;n\;even$
Multiquadratic (MQ)	$\sqrt{1+\epsilon^{2}r^{2}}$
Inverse multiquadratic (IMQ)	$\frac{1}{\sqrt{1+\epsilon^{2}r^{2}}}$
Inverse quadratic (IQ)	$\frac{1}{1+\epsilon^{2}r^{2}}$
Gaussian (GS)	$e^{-\epsilon^{2}r^{2}}$
Generalized multiquadratic (GMQ)	${\left(\epsilon^{2}r^{2}+R^{2}\right)}^{q}$

## Data Availability

Not applicable.
